# Spatial Distribution Patterns, Environmental Drivers, and Hotspot Dynamics of the European Rabbit on a Mediterranean Island: Implications for Conservation and Management

**DOI:** 10.3390/biology14030225

**Published:** 2025-02-20

**Authors:** Yiannis G. Zevgolis, Foto Konsola, Athanasia-Zoi Bouloutsi, Niki-Nektaria Douskou, Ioanna Emmanouilidou, Maria-Alexandra Kordatou, Anastasia Lekka, Maria-Eirini Limnioti, Maria Loupou, Despoina Papageorgiou, Michailia-Theodora Papamakariou, Eleni Tsiripli, Panagiotis Tzedopoulos, Christos Xagoraris, Alexandros D. Kouris, Panayiotis G. Dimitrakopoulos

**Affiliations:** 1 Biodiversity Conservation Laboratory, Department of Environment, University of the Aegean, 81132 Mytilene, Greece; fkonsola@gmail.com (F.K.); env20061@env.aegean.gr (A.-Z.B.); env20019@env.aegean.gr (N.-N.D.); env20020@env.aegean.gr (I.E.); env20032@env.aegean.gr (M.-A.K.); env22023@env.aegean.gr (A.L.); env19051@env.aegean.gr (M.-E.L.); env20043@env.aegean.gr (M.L.); env20069@env.aegean.gr (D.P.); env20071@env.aegean.gr (M.-T.P.); env22802@env.aegean.gr (E.T.); env21020@env.aegean.gr (P.T.); pdimi@aegean.gr (P.G.D.); 2 Department of Geography, School of Environment, Geography and Applied Economics, Harokopio University, 17671 Athens, Greece; cxago@hua.gr; 3 Department of Sustainable Agriculture, University of Patras, 30131 Agrinio, Greece; up1113010@upatras.gr

**Keywords:** *Oryctolagus cuniculus*, spatial distribution, spatial clustering, hotspots, spatial lag models, Lemnos Island, Greece

## Abstract

The European rabbit (*Oryctolagus cuniculus*) presents a significant conservation and management challenge in Greece. While it has been listed in national biodiversity assessments, its population dynamics on the island of Lemnos demonstrate the characteristics of a highly adaptable and rapidly expanding species, exerting substantial ecological and economic impacts. Addressing this issue requires a spatially explicit understanding of its distribution patterns and habitat preferences, particularly given its extensive population growth over the past three decades. To this end, we conducted 40 field surveys across the island, documenting 1534 presence records of the species. We applied Kernel Density Estimation, Getis-Ord Gi *, and Anselin Local Moran’s I to identify the spatial distribution patterns and significant hotspots. A spatial lag model was used to quantify hotspot intensity and clustering dynamics, while abiotic, biotic, and anthropogenic factors were analyzed to assess habitat associations. Our results revealed that rabbit hotspots are predominantly concentrated in fertile lowland agroecosystems, with nearly 60% of high-density areas overlapping conservation zones. Soil and field conditions, grazing-supporting landscapes, and arable and subsidized agricultural areas emerged as significant predictors of *O. cuniculus* presence. The observed spatial dependencies indicated that while hotspot intensities and clustering dynamics are influenced by the conditions in neighboring areas, habitat characteristics remain fundamental in shaping their distribution, highlighting the broader landscape-scale spatial patterns affecting rabbit populations. These findings underscore the necessity of adopting spatially informed management strategies that mitigate agricultural impacts while accounting for interconnected spatial dynamics, providing a foundation for informed decision-making to manage rabbit populations while balancing conservation and agricultural priorities.

## Simple Summary

The European rabbit is regarded as an agricultural pest on the island of Lemnos, Greece. Over the past three decades, its population on the island has rapidly expanded, resulting in significant ecological disruptions and economic losses. To gain a clearer understanding of its distribution and habitat preferences, we conducted field surveys across the island and utilized spatial analysis techniques to identify high-density areas (hotspots) and the environmental factors driving its presence. Our findings revealed that rabbits are highly concentrated in fertile lowland plains, particularly in areas with cultivated and abandoned fields. Notably, more than half of the high-density areas overlapped with conservation zones, and habitat preferences were strongly influenced by factors that support burrow construction, provide shelter, and ensure abundant food resources. Spatial modeling further revealed that habitat conditions in one area significantly affect rabbit densities in neighboring regions, emphasizing the interconnected nature of their populations. Our findings provide essential information to guide targeted management strategies that may balance agricultural needs with conservation priorities in insular environments.

## 1. Introduction

Species introductions beyond their native ranges, either through intentional or accidental means, have emerged as a significant catalyst for global biodiversity loss and ecosystem transformation in recent years [1,2], with particularly pronounced impacts observed in fragile ecosystems [3,4]. Among these, islands stand out as being exceptionally vulnerable to the effects of introduced species [5,6,7,8,9] due to their geographic isolation, evolutionary uniqueness, and ecological fragility [10,11]. This inherent vulnerability is further magnified by several interrelated ecological factors: high levels of species endemism [12], low species richness [13], reduced complexity in trophic relationships [14], scarcity or absence of predators [15], and the prevalence of native species [16,17], all of which collectively heighten the susceptibility of island ecosystems to severe disruptions, such as predation, competition, and herbivory, by introduced species [18,19,20,21].

On islands, the introduction of mammalian terrestrial predators such as red foxes (*Vulpes vulpes*) [22], black rats (*Rattus rattus*) [23], brown rats (*Rattus norvegicus*) [23], and domestic cats (*Felis catus*) [24,25] has been particularly detrimental, leading to ecosystem disturbances, significant declines in native species populations [26], and, in some cases, localized extinctions [27]. Compounding these predatory pressures, introduced herbivores such as feral goats (*Capra hircus*) [28], cattle (*Bos taurus*) [29], deer (Cervidae) [30], and European rabbits (*Oryctolagus cuniculus*) are recognized as some of the most ecologically disruptive taxa [31] due to their generalist feeding habits and the ability to thrive in diverse environmental conditions, which allow them to rapidly colonize and dominate island ecosystems [32]. As a result, many islands have been pushed beyond ecological tipping points, where natural recovery becomes exceedingly difficult, if not impossible [19].

Among the human-mediated introduced species, the European rabbit (*O. cuniculus*) stands out as one of the most widespread and ecologically disruptive herbivores in island ecosystems [31]. Native to the Iberian Peninsula, *O. cuniculus* has been deliberately introduced to over 800 islands worldwide [33], primarily for hunting, meat production, and the fur trade [34,35]. Its ability to establish populations in non-native environments [34] is largely attributed to a suite of biological traits, including a generalist diet, high grazing efficiency, reproductive potential, and the ability to construct burrows, which provide shelter and create microhabitats that facilitate survival in challenging conditions [36,37].

As selective herbivores, rabbits exert significant pressure on island ecosystems, primarily through the overgrazing and selective browsing of plant species [21,38,39,40] suppressing native vegetation regeneration and altering plant community dynamics [31,41]. This selective feeding behavior frequently promotes the dominance of less palatable or invasive plant species, further destabilizing native plant assemblages and disrupting ecological balance on island ecosystems [21,42]. Beyond such direct impacts on vegetation, rabbits also serve as ecosystem engineers, reshaping soil composition, nutrient cycling, and hydrological regimes [43,44]. Such alterations often trigger cascading ecological effects, disrupting habitat structures and resource availability for species dependent on intact vegetation for food, shelter, or reproduction [41]. Additionally, rabbits also impose significant economic burdens on agriculture, causing extensive crop damage and necessitating costly pest control measures [45,46,47,48].

This combination of biological adaptability and ecological impact has positioned the European rabbit as one of the most paradoxical species in conservation biology [34]. While globally recognized as Endangered on the IUCN Red List due to severe population declines in its native range [49], the species simultaneously ranks among the top 100 worst invasive species worldwide [50]. This dual status reflects its contrasting ecological roles; a keystone species in its native range [51,52] and an invasive herbivore causing substantial ecological and economic damage to the island ecosystems it invades.

In Greece, *O. cuniculus* presents a compelling example of an introduced species that has become ecologically established, particularly on the Aegean and Ionian islands where it was likely introduced centuries ago by Phoenician traders [33]. The species has now been included in national biodiversity assessments [53], yet its population dynamics on Lemnos Island have followed a different trajectory, with its numbers surging dramatically over the last three decades [46,54], resulting in severe ecological and economic impacts and leading the Greek authorities to categorize the species as an agricultural pest [46,55].

While this complexity underscores the challenges inherent in balancing conservation priorities with agricultural interests and highlights the complexity of managing *O. cuniculus* on Lemnos, effective decision-making necessitates a contemporary, spatially explicit understanding of the species’ distribution. Previous efforts to model the species distribution on the island in 2008 [46] offered valuable baseline data, but the continuous assessment and monitoring of its populations through time are necessary. Over the past 16 years, significant changes in the socioecological [56], and climatic conditions [57] have likely reshaped the species’ distribution patterns, habitat preferences, and population densities. These dynamic shifts, compounded by the absence of current fine-scale spatial data, including the identification of the species’ hotspots and localized areas of concern, challenge our ability to formulate targeted management strategies that effectively balance conservation priorities with the imperative of mitigating agricultural impacts. Therefore, the identification of the species’ spatial patterns is vital for determining the regions that require urgent intervention, whether to mitigate crop damage, prevent habitat degradation, or protect native species. Without such information, conservation measures risk being overly generalized, potentially overlooking localized impacts or misallocating resources, thus diminishing their effectiveness.

To address these challenges, our study focuses on Lemnos Island, Greece, aiming to (a) identify the key abiotic, biotic, and anthropogenic factors influencing the presence of *O. cuniculus*; (b) analyze its spatial distribution patterns to detect emerging hotspots, clusters, and spatial outliers, thereby providing essential information to guide targeted conservation strategies aimed at mitigating the impacts of the species on vulnerable habitats and native species; and (c) determine the underlying drivers of these observed spatial patterns, with a specific focus on the intensity of hotspots and the dynamics of spatial clustering.

## 2. Materials and Methods

### 2.1. Study Area

**Figure 1:**
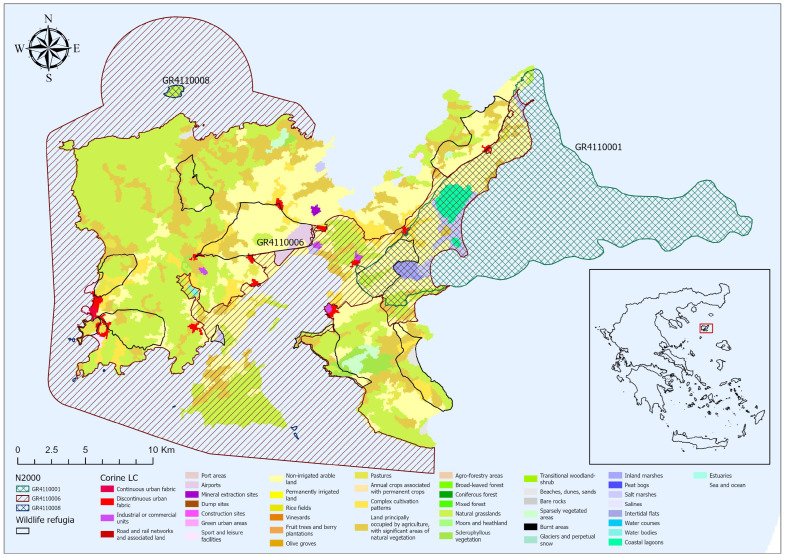
Distribution map of the main land habitats across the island of Lemnos. Key geographical features, including Natura 2000 sites and wildlife refugia, are highlighted.

Lemnos Island, located in the northeastern Aegean Sea, is the eighth largest island of Greece, covering an area of 476.6 km^2^ (Figure 1). Positioned approximately 30 km from the nearest island and 60 km from the continental coast, Lemnos exhibits a pronounced insular character due to its geographic isolation. Unlike most Aegean islands, which are typically characterized by rugged coastlines and steep mountainous terrain, Lemnos features a predominantly low-relief landscape, with its highest point reaching 430 m a.s.l. [58]. The western and northern regions are dominated by phrygana and short grasslands, which are often subject to intensive sheep and goat grazing [59]. In contrast, the central and eastern plains are fertile and support extensive agricultural mosaics, including vineyards, cereal fields, and livestock pastures [60].

The island also hosts semi-natural habitats with high ecological value, such as wetlands, lagoons, sand dunes, remnants of oak forests, and scrublands, which collectively enhance the biodiversity richness [58,61]. Approximately 26.5% of Lemnos’ terrestrial area (126.6 km^2^) is included in the Natura 2000 network (sites GR4110001, GR4110006, and GR4110008) [62], encompassing a range of critical habitats such as coastal marshes, brackish lakes, seasonal wetlands, and sand dune systems [63]. Additionally, 11 designated wildlife refugia, covering approximately 109 km^2^, contribute further to habitat conservation. While some of these wildlife refugia overlap partially with Natura 2000 sites (Figure 1), they collectively form a conservation network essential for preserving the island’s unique ecological features.

The climate of Lemnos is classified as meso-Mediterranean, characterized by hot, dry summers and mild, wet winters, with an average annual temperature of 16.1 °C and an average annual precipitation of 502.7 mm/year [64].

### 2.2. Field Surveys

To assess the spatial distribution of *O. cuniculus*, we employed a randomized grid-based approach to ensure comprehensive and unbiased coverage of the island’s landscape. Using ArcGIS 10.7 software (ESRI Inc., Redlands, CA, USA), we divided the island into 165 grid cells (2 × 2 km), with most measuring 4 km^2^, while some were smaller due to adjustments being required to align with the island’s irregular geographical boundaries.

We conducted a total of 40 field surveys over a 10-week period from mid-June to late August 2023, covering the entire island. This period was strategically chosen based on previous studies [46] to coincide with post-harvest conditions, which improved visibility and access to agricultural fields. Additionally, this period followed the breeding season and preceded the hunting season, reducing anthropogenic disturbances and maximizing the likelihood of detecting the species. Each cell was visited once, and the order of visitation was randomized to minimize biases associated with habitat type, terrain accessibility, or temporal variation in the species’ activity. To achieve this, each grid cell was assigned a unique identifier, and the visitation sequence was pre-generated in a random manner before fieldwork commenced. The randomization process was executed by generating a shuffled list of grid cell identifiers, ensuring that each grid cell had an equal probability of selection.

Randomization was also applied across days and survey routes, ensuring an even distribution of survey effort by avoiding the over-representation of specific habitats. Survey days were randomly assigned by selecting grid cells from the pre-generated list without imposing any spatial constraints, meaning that adjacent grid cells could be visited sequentially if selected by chance. Additionally, the survey routes were determined each day by randomly selecting an access point and a travel direction within each grid cell to further minimize observer biases.

The surveys were conducted four days per week during the early morning (thirty minutes before sunrise to approximately four hours after sunrise) and the late afternoon to early night (two hours before sunset to three hours after sunset). These time windows align with the crepuscular–nocturnal activity patterns of *O. cuniculus* [65,66,67], when its movement peaks and environmental conditions are most favorable for visual detection [68]. Midday surveys, when high temperatures typically suppress the species’ activity, as well as days with adverse weather conditions (e.g., heavy rain, strong winds) were avoided to maintain observation reliability.

We employed a combination of vehicle-based and on-foot surveys to adapt to varying habitat types, terrain accessibility, and visibility conditions across grid cells. Vehicle-based surveys were conducted along accessible primary and secondary road networks at a controlled speed of 10–20 km/h. During these surveys, a team of two researchers used binoculars for daytime observations and spotlights at night. In contrast, on-foot surveys were conducted in areas inaccessible by vehicles, following natural trails while scanning the surrounding habitat for the species’ presence. Observer interference was minimized by adhering to standardized survey protocols, including maintaining a consistent low survey speed, reducing noise levels during on-foot surveys, and using unobtrusive observation methods (binoculars and spotlights) to reduce direct human influence on rabbit behavior.

Throughout all surveys, only direct sightings of *O. cuniculus* were recorded to ensure methodological consistency and avoid potential biases associated with indirect signs such as tracks, droppings, or burrows. Each observation was georeferenced using handheld GPS devices and accompanied by metadata, including time of observation, habitat type, and weather conditions.

### 2.3. Abiotic, Biotic, and Anthropogenic Metrics

To identify the key abiotic, biotic, and anthropogenic factors influencing the presence of *O. cuniculus* on the island, we extracted and analyzed a set of 31 spatial variables using ArcGIS 10.7 (ESRI Inc., Redlands, CA, USA). For this, we created circular plots with a radius of 50 m centered on each recorded presence point, representing an ecologically relevant spatial scale based on the average home range estimates from previous studies [68,69,70]. Within each 50 m circular plot, we initially extracted key abiotic metrics which are known to influence burrow construction, predator avoidance, and thermoregulation [71]. These included elevation (minimum, maximum, mean) and slope (minimum, maximum, mean) which were derived from a 10 × 10 m Digital Elevation Model (DEM) provided by the Biodiversity Conservation Laboratory, University of the Aegean. Additionally, we included soil depth, an essential factor affecting burrow stability and vegetation productivity, which was sourced from the ISRIC SoilGrids dataset with a 250 × 250 m spatial resolution [72].

Thereafter, we extracted key biotic metrics to characterize vegetation structure, productivity, and land cover composition, all of which are essential determinants of the *O. cuniculus* habitat preferences. Vegetation productivity (mean, minimum, maximum) was assessed for the months corresponding to the survey period using high-resolution total productivity data from the Copernicus Vegetation Productivity Dataset [73]. To capture the fine-scale vegetation structure, we included grasslands and small woody feature cover, derived from Copernicus High-Resolution Layers [74] at a 10 × 10 m resolution. Grasslands are known to be a primary foraging habitat for *O. cuniculus*, while small woody features provide shelter and aid in predator avoidance [34]. Additionally, non-tree areas, derived from the Copernicus Forest Type Dataset [75], were included to represent open habitats favored by rabbit populations due to the improved visibility and reduced predation risk [44]. Land-use composition was quantified using datasets from CORINE Land Cover [76] and the Greek Payment Authority of Common Agricultural Policy Aid Schemes (OPEKEPE) [77]. From these datasets, we distinguished two key variables: the total agricultural cover derived from the CLC dataset, which reflects broad European land-use classifications, and the agricultural land cover from the OPEKEPE dataset, which provides more localized and detailed information on actively managed agricultural areas in Greece. We also extracted the percentage cover for arable land, pastures, vineyards, and olive groves, and we calculated the extent of both agricultural land meeting the European Union’s sustainability standards (good agricultural and environmental conditions—GAEC), and agricultural landscapes receiving support measures through state-backed programs (state-supported agricultural areas).

To evaluate the potential influence of human activities on the species’ habitat preferences, we extracted and calculated several anthropogenic factors, including proximity to roads and settlements. Furthermore, to incorporate the potential regulatory and protective influence of conservation areas, we also included the proximity to Natura 2000 sites and to designated wildlife refugia as key metrics. In addition, we recorded whether each circular plot overlapped with the boundaries of Natura 2000 sites (GR4110001 and GR4110006) or wildlife refugia, and we calculated the percentage of each circular plot falling within these conservation areas. These datasets were sourced from the open platform of geospatial data and services for Greece (geodata.gov.gr (accessed on 15 November 2024)).

### 2.4. Spatial Analysis

To investigate the spatial distribution patterns of *O. cuniculus* and to explore both large-scale patterns and localized anomalies, we applied a suite of spatial techniques designed to identify density trends, emerging hotspots, spatial clusters, and potential outliers. We initially used the Kernel Density Estimation (KDE), a non-parametric statistical technique widely used for estimating the probability density function of spatial point patterns [78,79], to estimate the underlying probability density of the species across the island. We employed a kernel function that weighted the surrounding points based on their proximity to confirmed records to account for spatial clustering and ensure unbiased density estimates. Moreover, the bandwidth was set at 50 m based on ecological considerations to represent the average home range radius of *O. cuniculus* [68,69,70].

Next, in order to detect regions of clustering, hotspots, and spatial outliers, we applied two complementary spatial statistical techniques: Getis-Ord Gi * and Anselin Local Moran’s I. The term “hotspot” has been widely used across disciplines to describe areas exhibiting relatively higher values compared to their surroundings [80,81,82,83,84]. Here, we define hotspots as locations demonstrating statistically significant clustering (local autocorrelation) in the spatial distribution of *O. cuniculus* presence records.

We applied the Getis-Ord Gi * statistic [85,86] to identify regions where the observed spatial clustering deviates significantly from a random spatial distribution, determining whether high or low species presence records are spatially concentrated beyond what would be expected by chance. We used a fixed distance band of 50 m for calculating neighborhood statistics; this distance band resulted in high z-score values as an indication of the clustering patterns in the presence data. This analysis generates z-scores (GiZscore) and *p*-values, which are essential for interpreting the statistical significance and direction of clustering [87]. Positive z-scores with significant *p*-values represent hotspots (intense clustering of high values), indicating areas where rabbit presence is significantly higher than expected, while negative z-scores with significant *p*-values represent coldspots (intense clustering of low values), reflecting areas where rabbit presence is significantly lower than expected.

To complement the hotspot analysis and examine the localized spatial relationships, we applied Anselin Local Moran’s I statistic [86]. This method identifies spatial clusters of similar values (High–High or Low–Low) as well as spatial outliers (High–Low or Low–High) through the calculation of Local Moran’s I z-scores (LMiZscores), providing a finer scale perspective on the spatial arrangement of *O. cuniculus* presence records.

### 2.5. Statistical Analysis

To enable a robust comparison between locations with and without *O. cuniculus*, we randomly generated pseudo-absences using ArcGIS 10.7 (ESRI Inc., Redlands, CA, USA). The use of pseudo-absences was essential due to the inherent challenges of confidently identifying true absences for such a mobile species, particularly in habitats where detectability is influenced by vegetation density, terrain complexity, or limited accessibility. To ensure the ecological plausibility of the pseudo-absence locations, each point was randomly placed across the study area while maintaining a minimum distance of 100 m from any recorded presence point. This distance, double the 50 m radius used for the presence point circular plots, minimized the risk of spatial overlap and reduced the potential biases stemming from proximity to known presence records. The number of pseudo-absences was set to match the number of presences [88], ensuring a balanced dataset for subsequent statistical analysis. For each pseudo-absence, we mirrored the same extraction and calculation procedures applied to the presences, quantifying abiotic, biotic, and anthropogenic metrics within a 50 m circular plot centered on each pseudo-absence. To standardize the datasets for statistical analysis, all extracted continuous variables were transformed into z-scores.

We then applied a principal component analysis (PCA) in R statistical environment (v. 4.4.2, R Core Team, Vienna, Austria) [89] to address multicollinearity among the variables and reduce dimensionality [90]. Before performing the PCA, we ensured dataset suitability by examining the Kaiser–Meyer–Olkin (KMO) measure of sampling adequacy and performing Bartlett’s test of sphericity. A KMO value between 0.5 and 1.0 confirmed that sampling adequacy was sufficient for reliable component extraction, while Bartlett’s test ensured that the dataset displayed significant correlations suitable for PCA. Variables with low communality values (less than 0.6), indicating a limited contribution to the retained components, were excluded to improve the analytical robustness. Principal components with eigenvalues greater than 1.0 were retained using the Varimax rotation method, enhancing interpretability by maximizing the variance explained by each extracted component. The PCA was performed using the “FactoMineR” package in R.

The extracted principal components were subsequently used as predictor variables in a binary logistic regression (BLR) model conducted in R (v. 4.4.2, R Core Team, Vienna, Austria) using the “glm” function with a logit link function, where the presence–absence dataset served as the dependent variable. Model performance was assessed using Nagelkerke’s R^2^ [91], while goodness-of-fit was evaluated through the Hosmer–Lemeshow test. The model’s discrimination ability was analyzed using a classification table comparing observed versus predicted values. Additionally, the area under the Receiver Operating Characteristic (ROC) curve was employed to quantify the model’s predictive accuracy and identify the optimal probability threshold for classifying presence and absence records [92]. The “pROC” package in R was used to compute AUC values and visualize model performance. To further interpret the contribution of each predictor variable, we conducted Relative Importance Analysis (RIA), which quantified the proportional contribution of each principal component to the overall predictive performance of the BLR model. This analysis, based on standardized coefficients and likelihood-based metrics, ranked the predictors in terms of their relative influence, providing insights into which factors most strongly impacted *O. cuniculus* presence. To complement these analyses, we generated effect plots for each principal component using the “effects” package in R to visualize their influence on the probability of *O. cuniculus* presence.

Finally, to evaluate whether the principal components derived from the PCA could explain the spatial distribution patterns of *O. cuniculus*, specifically (a) the intensity of hotspot formation and (b) the clustering dynamics, controlling for the spatial autocorrelation inherent in the data, we employed a spatial lag model (SLM) using GeoDa software (v. 1.22.0.10) [93]. The SLM is a spatial regression approach commonly used to analyze spatial patterns in ecological datasets [94,95,96,97] by accounting for spatial autocorrelation through the inclusion of a spatial lag coefficient (Rho). This spatial autoregressive parameter allows the dependent variable at each location to be influenced not only by the independent variables at that location but also by the dependent variable values of neighboring locations, thus addressing potential spatial spillover effects. In this context, spatial spillover effects refer to the phenomenon where the spatial patterns observed in one location extend beyond their immediate boundaries, influencing adjacent areas through ecological and landscape-level interactions. In the SLM framework, this effect is statistically captured when the Rho coefficient is significant, indicating that variations in the dependent variable are not solely driven by local predictors but also by interactions across spatially adjacent locations [93,96]. The Rho coefficient quantifies the degree of spatial dependence in the model by measuring the extent to which neighboring observations influence local outcomes. A high and statistically significant Rho value indicates that species distributions are not spatially independent but instead follow a structured pattern shaped by spatial dependencies with adjacent areas.

The Getis-Ord Gi z-scores (GiZscore) and Local Moran’s I z-scores (LMiZscore) were used as dependent variables to assess hotspot intensity and clustering dynamics, respectively. The extracted principal components served as independent predictor variables, while the inclusion of a spatial lag coefficient accounted for the influence of neighboring clustering patterns.

## 3. Results

### 3.1. Spatial Distribution of Oryctolagus cuniculus on Lemnos Island

**Figure 2:**
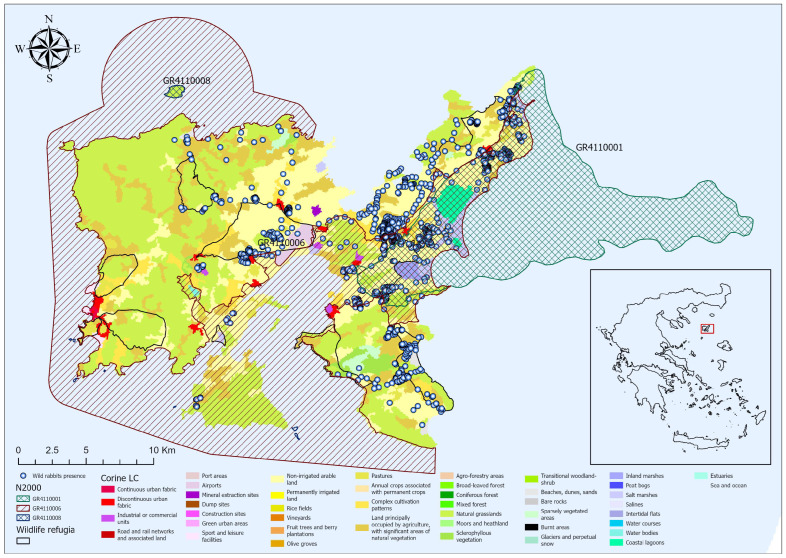
Spatial distribution of *Oryctolagus cuniculus* presence records across Lemnos Island, Greece. The map highlights land cover categories based on CORINE Land Cover (CLC) classification and includes key geographical features such as Natura 2000 sites (GR4110001, GR4110006) and designated wildlife refugia.

We documented a total of 1534 *O. cuniculus* presence records across Lemnos Island, providing a comprehensive dataset to evaluate the species’ habitat preferences and spatial patterns (Figure 2). A higher concentration of the species was documented in the central and eastern regions, where fertile agricultural plains dominate. In contrast, fewer records were documented in the western and northern areas, and they were characterized by phrygana and natural grasslands. Similarly, a few records were noted in the southern and southeastern coastal zones.

The species predominantly occupied low-elevation areas (30.3 ± 24.3 m) with shallow gradients, reflected in a mean slope of 2.3 ± 2.2% and soils with substantial depth (199.9 ± 1.2 cm). Proximity to anthropogenic features varied, with *O. cuniculus* exhibiting a mean distance of 77.2 ± 69.2 m to roads and 1067.6 ± 568.4 m to settlements.

In terms of habitat usage, the majority of records were in non-irrigated arable land, which accounted for 57.4% of the total records, followed by complex cultivation patterns, representing 18.8% of the records. Natural grasslands and phrygana accounted for only 3.2% and 0.2% of records, respectively, while a substantial proportion of them overlapped with conservation areas. Specifically, 738 records (48.1%) were within Natura 2000 sites, while 322 (21%) were found within designated wildlife refugia.

### 3.2. Principal Components of Abiotic, Biotic, and Anthropogenic Metrics

The PCA identified five significant components with eigenvalues exceeding 1, cumulatively explaining 87.59% of the total variance in the dataset (Table 1). The first component (PC_1_—elevation gradient), with an eigenvalue of 4.687, explained 36.05% of the total variance and was strongly associated with elevation metrics (minimum, maximum, and mean elevation). The second component (PC_2_—arable and subsidized agriculture), with an initial eigenvalue of 2.391, accounted for 18.39% of the variance and was driven by cereal crop cover, agricultural land cover, and state-supported agricultural area. The third component (PC_3_—productivity potential), with an initial eigenvalue of 2.122, contributed 16.32% of the variance and was dominated by productivity metrics. The fourth component (PC_4_—grazing-supporting landscapes), with an initial eigenvalue of 1.119, explained 8.61% of the total variance, with total agricultural cover and pasture cover as the dominant variables. Finally, the fifth component (PC_5_—soil and field conditions), with an initial eigenvalue of 1.068, accounted for 8.22% of the variance, being strongly associated with good agricultural and environmental conditions (GAEC) and soil depth.

### 3.3. Modeling the Presence of Oryctolagus cuniculus on Lemnos Island

The BLR model retained all five PCs identified through PCA as significant predictors of the presence of *Oryctolagus cuniculus* on Lemnos Island. The model exhibited a robust statistical performance, (χ^2^ (5, N = 3068) = 2850.50, *p* < 0.001; Table 2), confirming the collective influence of these predictors. The Nagelkerke R^2^ indicated that the model explained 81.3% of the variance in rabbit presence, while the Hosmer–Lemeshow test showed that the model’s goodness of fit can be accepted (Hosmer–Lemeshow = 674.98, *p* > 0.05) due to the absence of Chi-square significance.

The area under the ROC curve (AUC = 0.967, S.E. = 0.001, 95% CI 0.961–0.974, *p* < 0.001), as a metric of the sensitivity values for all possible values of specificity, with a threshold (0.699) resulting from Youden’s index [98] and achieving an overall classification accuracy of 94.3%, correctly identified 95.3% of absences and 93.3% of presences, illustrating its high discriminative ability.

Soil and field conditions (PC_5_) emerged as the most influential variable, with a strong positive association with rabbit presence (B = 4.404, *p* < 0.001). Similarly, arable and subsidized agricultural areas (PC_2_) and productivity potential (PC_3_) positively influenced rabbit presence (PC_2_: B = 1.497, *p* < 0.001; PC_3_: B = 1.023, *p* < 0.001). In contrast, elevation gradient (PC_1_: B = −2.123, *p* < 0.001) and grazing-supporting landscapes (PC_4_: B = −2.519, *p* < 0.001) had a significant negative impact on rabbit presence.

The Relative Importance Analysis (RIA) highlighted the varying contributions of each predictor variable in the BLR model. Among the variables, soil and field conditions (PC_5_) emerged as the most influential predictor, accounting for 57.84% of the model’s relative importance (z = 12.49, *p* < 0.001). The grazing-supporting landscapes (PC_4_) contributed the most substantial negative effect, representing −18.92% of the relative importance (z = −8.29, *p* < 0.001), followed by the elevation gradient (PC_1_) with −13.43% (z = −7.30, *p* < 0.001). Positive contributions were observed for arable and subsidized agricultural areas (PC_2_) at 6.68% (z = 7.64, *p* < 0.001) and productivity potential (PC_3_) at 3.12% (z = 5.82, *p* < 0.001).

**Figure 3:**
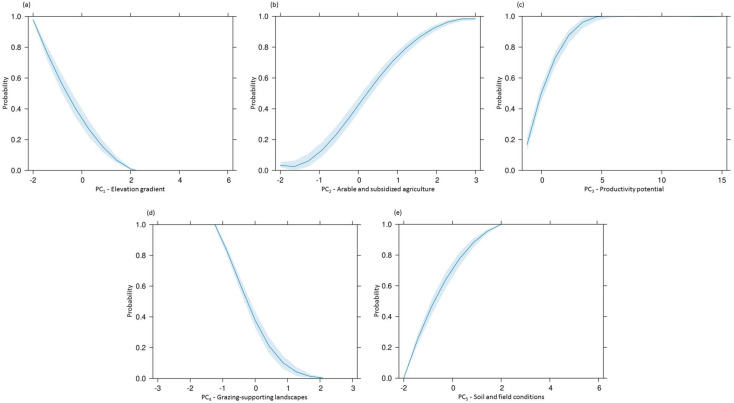
Effect plots of the impact of (**a**) PC_1_—elevation gradient, (**b**) PC_2_—arable and subsidized agriculture, (**c**) PC_3_—productivity potential, (**d**) PC_4_—grazing-supporting landscapes, and (**e**) PC_5_—soil and field conditions, on the presence of *Oryctolagus cuniculus* on Lemnos Island. The x-axis represents the PCs, while the y-axis displays the corresponding probability.

To complement these findings, effect plots were utilized to visually illustrate the impact of the most influential variables on *O. cuniculus* presence. These plots provide a clear representation of how changes in predictor variables influence the probability of the species occurrence. These visualizations (Figure 3) complement the RIA, enabling a comprehensive understanding of the direction and magnitude of each variable’s effect.

### 3.4. Spatial Patterns and Hotspot Areas of Oryctolagus cuniculus

**Figure 4:**
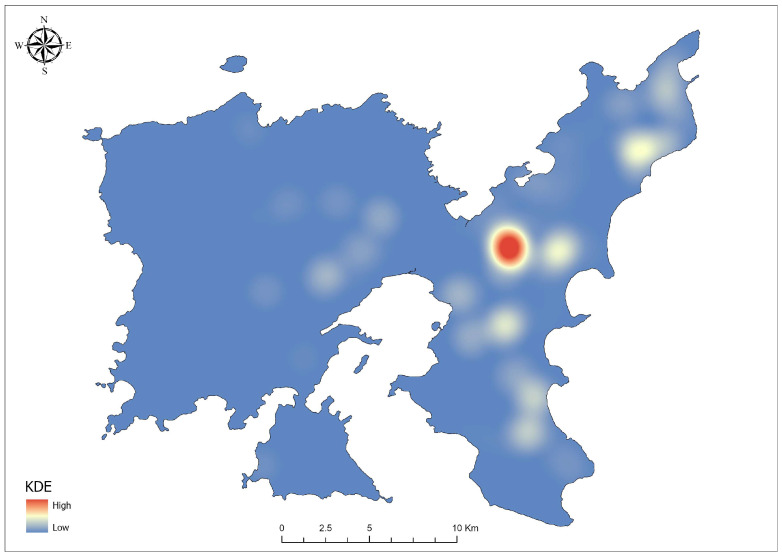
Kernel Density Estimation (KDE) map illustrating the spatial distribution of *Oryctolagus cuniculus* presence across Lemnos Island. High-density areas (red) indicate regions with concentrated presences, while low-density areas (blue) represent regions with fewer records.

The KDE analysis provided a spatially detailed representation of the distinct distribution patterns of *O. cuniculus*, emphasizing the high-density areas with concentrated presence records across Lemnos Island (Figure 4). These high-density zones, primarily localized in the central and northeastern region, indicate key habitats where environmental and anthropogenic factors align to support robust rabbit populations.

**Figure 5:**
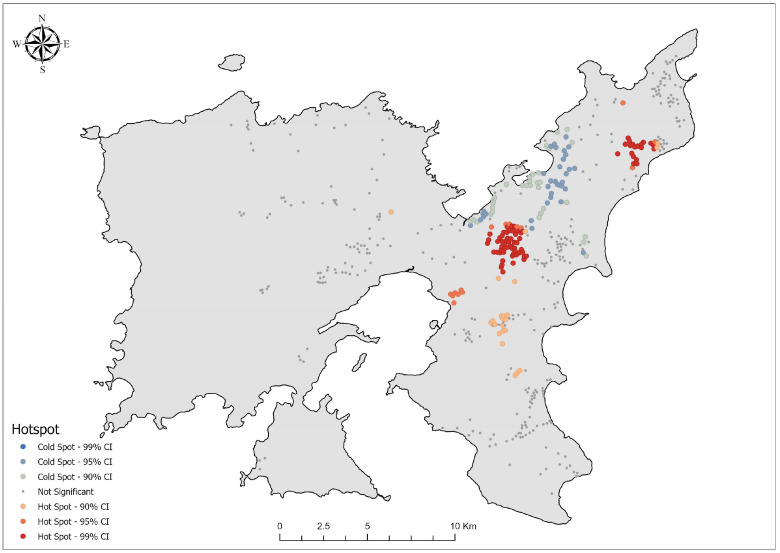
Hotspot analysis map of *Oryctolagus cuniculus* presence on Lemnos Island using the Getis-Ord Gi * statistic. Hotspot regions are categorized by confidence levels (99%, 95%, and 90%) and displayed in red gradients, while coldspot regions are similarly categorized and displayed in blue gradients. Non-significant areas are shown in gray.

The Getis-Ord Gi * analysis revealed the significant spatial clustering of *O. cuniculus* presence across Lemnos Island, categorized into hotspots, coldspots, and non-significant areas based on confidence levels (Figure 5). Significant hotspot areas, defined by z-scores, were predominantly concentrated in the central and northeastern regions of the island. A total of 72 hotspots were identified at the 99% confidence level (z-score > 2.58), encompassing 460 records, with z-scores ranging from 2.75 to 8.33 (mean = 5.16, SD = 1.37). Of these, 43 hotspots were located within Natura 2000 sites.

At the 95% confidence level (1.96 < z-score ≤ 2.58), fifteen hotspots were detected, nine of which were situated within Natura 2000 sites, containing a total of 74 records. The z-scores for these hotspots ranged from 2.01 to 2.52 (mean = 2.19, SD = 0.08). Additionally, 22 moderate hotspots at the 90% confidence level (1.65 < z-score ≤ 1.96) were identified, accounting for 89 records. These hotspots exhibited z-scores ranging from 1.68 to 1.90 (mean = 1.78, SD = 0.06).

In contrast, coldspots, representing areas with significantly low concentrations of *O. cuniculus*, were predominantly observed in the northeastern parts of the island, between the main hotspot areas. At the 90% confidence level (−1.96 ≤ z-score < −1.65), 32 moderate coldspots were detected, containing 37 records, with z-scores ranging from −1.95 to −1.68 (mean = −1.84, SD = 0.09). Similarly, 34 coldspots were identified at the 95% confidence level (−2.58 ≤ z-score < −1.96), encompassing 34 records, with z-scores ranging from −2.43 to −1.96 (mean = −2.12, SD = 0.10). Among these, seven coldspots were located within Natura 2000 sites.

Non-significant areas, representing neutral spatial patterns with no significant clustering, covered a large portion of the island. These areas comprised 328 neutral spots, containing a total of 840 records. The z-scores in these regions ranged from −1.65 to 1.65 (mean = 0.27, SD = 0.87).

### 3.5. Identification of Significant Clustering Patterns of Oryctolagus cuniculus Distribution

**Figure 6:**
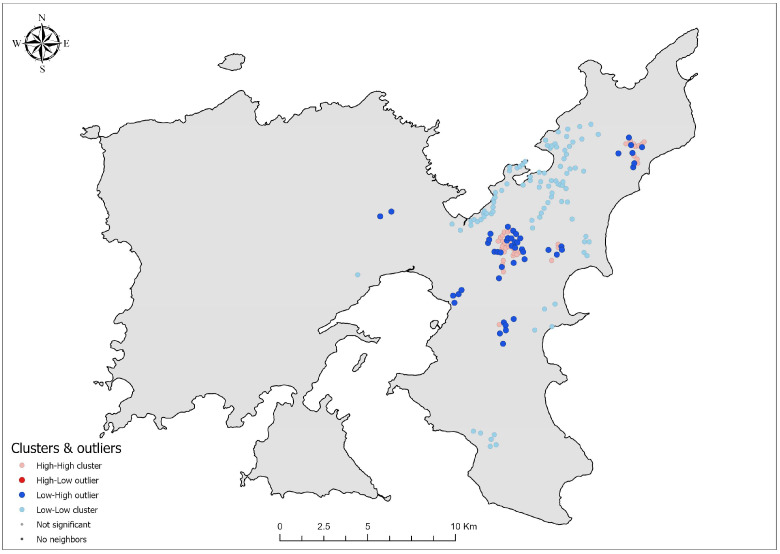
Anselin Local Moran’s I analysis of *Oryctolagus cuniculus* on Lemnos Island. The map categorizes clusters into High–High (HH) clusters, where high presence values are surrounded by other high values; Low–Low (LL) clusters, where low values are surrounded by other low values; and Low–High (LH) outliers, where low values are surrounded by high values.

Anselin Local Moran’s I analysis revealed the distinct spatial clustering patterns of *O. cuniculus* presence on Lemnos Island, identifying High–High (HH), Low–Low (LL), and Low–High (LH) clusters (Figure 6). High–High (HH) clusters, representing areas where high presence values are surrounded by other high values, emerged as the most prominent spatial pattern. A total of 37 HH clusters were identified, encompassing 416 records. Of these, 25 clusters were located within Natura 2000 sites. Local Moran’s I z-scores for HH clusters ranged from 1.93 to 8.46, with a mean of 5.21 (SD = 2.06), indicating the significant aggregation of records in the central and northeastern parts of the island.

Low–Low (LL) clusters, indicative of areas where low presence values are surrounded by other low values, were also significant. A total of 91 LL clusters were detected, containing 102 records, with 19 clusters situated within Natura 2000 sites. Local Moran’s I z-scores for LL clusters ranged from 0.98 to 2.25 (mean = 1.51, SD = 0.24). These clusters were primarily located in the southern and northeastern regions of the island (Figure 6).

Low–High (LH) clusters, representing spatial outliers where low values are surrounded by high values, were less frequent but notable. A total of 46 LH clusters were identified, encompassing 78 records. Of these, 31 clusters were located within Natura 2000 sites. Local Moran’s I z-scores for LH clusters ranged from −7.89 to −1.99, with a mean of −3.82 (SD = 1.79), highlighting isolated low-density areas amidst high-density regions.

### 3.6. Factors Influencing Hotspot Intensity and Clustering Dynamics

The spatial lag model (SLM) identified significant spatial dependence, highlighting the influence of neighboring areas in shaping the intensity and distribution of *O. cuniculus* hotspots and clustering dynamics on Lemnos Island. The spatial lag coefficient (Rho = 0.6396, *p* < 0.001) revealed that hotspot intensity at a given location is statistically associated with clustering patterns in adjacent areas. The model exhibited robust performance, as indicated by the Akaike Information Criterion (AIC = 5237.42), Schwarz Criterion (SC = 5269.40), and a Likelihood Ratio Test (*p* < 0.001), confirming the presence of significant spatial dependence.

The SLM achieved a high explanatory power, accounting for 83.3% of the total variance (R^2^ = 0.833). Notably, 40.9% of the explained variance was attributed to the spatial lag coefficient, emphasizing the critical role of spatial dependence, while the remaining 42.4% was explained by environmental predictors (PC_2_–PC_5_). Among these, PC_2_ (arable and subsidized agriculture) emerged as the most influential predictor, showing a positive relationship with hotspot intensity (B = 0.154, *p* < 0.001) and contributing 12.5% to the total variance. In contrast, PC_4_ (grazing-supporting landscapes) exhibited the strongest negative effect (B = −0.212, *p* < 0.001), accounting for 17.3% of the variance. PC_3_ (productivity potential) also negatively influenced hotspot intensity (B = −0.082, *p* < 0.001), explaining 6.6% of the variance, whereas PC_5_ (soil and field conditions) contributed positively (B = 0.073, *p* = 0.014), explaining 6.0% of the variance (Table 3a).

When assessing the clustering dynamics based on Local Moran’s I z-scores, the SLM similarly revealed a significant spatial dependence (Rho = 0.6673, *p* < 0.001), further supporting the role of neighboring spatial patterns in shaping clustering dynamics.

Diagnostic metrics, including an AIC of 5475.47 and SC of 5507.45, confirmed the strong performance of the model, with the Likelihood Ratio Test (*p* < 0.001) validating the significant spatial dependence. The model explained 85.17% of the total variance in Local Moran’s I z-scores. Of this, 44.5% was attributed to the spatial lag coefficient, indicating the pervasive influence of spatial dependence. The remaining 40.67% of the variance was explained by environmental predictors (PC_2_–PC_5_). Consistent with the analysis of hotspot intensity, PC_2_ (arable and subsidized agriculture) emerged as the most significant predictor, positively influencing clustering intensity (B = 0.084, *p* < 0.01) and contributing 10.8% to the total variance. PC_5_ (soil and field conditions) also demonstrated a positive effect (B = 0.081, *p* < 0.001), explaining 10.5% of the variance. Conversely, PC_3_ (productivity potential) had a moderately negative impact (B = −0.073, *p* < 0.01), contributing 9.5% to the variance, while PC_4_ (grazing-supporting landscapes) showed a weaker negative effect (B = −0.076, *p* < 0.05), accounting for 9.8% of the variance (Table 3b).

## 4. Discussion

In this study, we presented a holistic and spatially multidimensional approach to investigate the spatial distribution, clustering dynamics, and environmental determinants influencing the presence of *Oryctolagus cuniculus* on Lemnos Island. By combining advanced spatial techniques with a randomized grid-based field survey design, we identified the spatial patterns of the species and elucidated the key habitat associations, while explicitly accounting for the spatial dependencies that influence hotspot intensity and clustering dynamics.

In parallel, the explicit integration of conservation areas into the analysis provided valuable perspectives into the complexities of managing an introduced species that has established extensive populations and poses significant challenges for agricultural and ecological management on Lemnos. This complexity is starkly highlighted by the finding that nearly 60% of the most significant hotspots (99% confidence level) overlap with Natura 2000 sites, as well as by the economic burden caused by the species’ presence on the island. After all, from 2007 to 2022, the Hellenic Organization of Agricultural Insurances (ELGA) [99] has disbursed over 7 million euros in compensation for crop yield losses caused by *O. cuniculus* on the island, averaging more than 450,000 euros annually [99]. These figures underscore the pressing need for spatially informed, ecologically sensitive strategies that address the species’ impacts on natural and agricultural landscapes on the island.

Exacerbating this dynamic are the unique socioecological conditions of Lemnos, shaped largely by its geographic isolation and the abandonment of agricultural land since the 1980s. Situated far from the continental coast and neighboring Aegean islands, Lemnos stands as a continental-shelf island that was once connected to the mainland and other adjacent landmasses during the Late Pleistocene, when sea levels were much lower [100,101]. This historical connection allowed it to retain a diverse array of species, including those with limited dispersal capabilities [102]. However, unlike many other continental-shelf islands, Lemnos exhibits significant ecological gaps, most notably the complete absence of terrestrial predators [46]. Although a relative predation pressure exists from birds of prey and domestic cats (pers. obs.), their impacts are generally limited due to the low predator density, non-specialized hunting behaviors, and the ability of rabbits to mitigate predation through behavioral adaptations [103,104] and high reproductive rates [37].

This predator-relaxed environment has profound implications for the island’s ecological dynamics, differentiating Lemnos from both the native range of *O. cuniculus* [105], and regions where it has been introduced, including the UK [34], Australia [106], New Zealand [107,108], and Chile [109]. In these areas, *O. cuniculus* serves as a critical prey item for terrestrial predators, which exert regulatory pressures on its population. In contrast, the absence of such predators on the island has facilitated unrestrained population growth, mirroring the ecological disruptions observed on isolated oceanic islands where introduced mammals frequently disturb local ecosystems [29,40]. This ecological imbalance has been further exacerbated by human-induced changes, including relatively recent demographic shifts [60] that led to the abandonment of agricultural land and the transition from traditional to modern farming practices.

Consequently, these interconnected factors have created a highly favorable environment for the expansion of *O. cuniculus* on Lemnos Island. This is exemplified by the high number of records obtained during our field surveys, representing the largest and most detailed spatial dataset of the species, not only for the island but also for Greece. Each record represents a distinct instance of direct observation; however, it is important to note that our survey design does not account for factors such as individual movement behaviors [110], seasonal variations [66], or overlapping home ranges [111], which could influence the distribution patterns presented in this study. Moreover, the inherent challenges posed by the biology of the species [37,110,112,113,114], coupled with their crepuscular–nocturnal activity, likely resulted in a reduced detection probability during our field surveys.

However, our decision to focus exclusively on crepuscular windows rather than extending into nocturnal time windows was informed by established methods in the Mediterranean region, where dusk and dawn counts are considered highly effective for estimating the species abundance [115,116,117], as more than half of the individuals are simultaneously aboveground during these periods, making them particularly suitable for monitoring [118,119]. Moreover, even during the hours before or after these time windows, the detectability of the species remained quite satisfactory due to the absence of predation, allowing rabbits to spend extended periods outside their burrows, engage in reduced anti-predator behavior [120,121], and exhibit a weaker midday resting phase (pers. obs.). This aligns with broader findings on mammal responses to predation risk, which can drive behavioral adaptations under selective pressure [122], mirroring the patterns observed in urban environments [123,124] and in other mammalian species, which often exploit predator-free niches [79].

These behavioral adaptations reflect how ecological conditions influence habitat use, creating a framework for understanding the environmental and anthropogenic drivers analyzed through PCA. The resulting components highlight the intricate interplay of topography, human-modified landscapes, vegetation productivity, and soil conditions in shaping *O. cuniculus* habitat preferences on Lemnos Island.

In this light, the BLR model allowed us to quantify the influence of the identified components, enabling a detailed evaluation of the probability of rabbit presence and the relative importance of each component in explaining this likelihood. The positive association and strong contribution of PC_5_ (soil and field conditions) emphasizes the role of underlying abiotic factors in shaping rabbit presence (Table 2, Figure 3). The deep alluvial soils found in the relatively flat central and eastern regions of Lemnos not only facilitate burrowing, but also support the island’s agricultural productivity. Soil depth, in particular, is a key determinant of warren suitability, as deeper soils are easier to excavate [125,126,127] and offer greater permeability to water [128]. Such soil characteristics enhance both the structural integrity and the microclimatic stability of warrens, making these areas highly favorable for rabbits [126]. Similarly, PC_2_ (arable and subsidized agriculture) and PC_3_ (productivity potential) exhibited strong positive associations with rabbit presence, highlighting the critical role of human-modified landscapes and vegetation productivity in shaping habitat preferences. PC_2_ reflects the influence of arable land and state-supported agricultural areas, where government subsidies or incentives promote the cultivation of crops like cereals and legumes [129,130]. Complementing this, PC_3_ emphasizes the significance of vegetation growth and habitat productivity. Areas with high productivity potential offer ample forage opportunities and support the nutritional needs of the species [131].

Conversely, PC_4_ (grazing-supporting landscapes) and PC_1_ (elevation gradient) exerted significant negative effects on the species presence. The negative association with PC_4_ can be attributed to land-use practices associated with semi-natural grasslands and grazing [60], including pastures where livestock and wild herbivores, such as the fallow deer (*Dama dama*), graze freely (pers. obs.). These practices encompass the early-season grazing of cereal fields and late-season grazing of stubble fields [60], leading to a reduction in available shelter and food resources, making these areas less suitable for *O. cuniculus*. In parallel, the seasonal movement of grazing animals across different fields and pastures further contributes to the diminishing quality of *O. cuniculus* habitats, thereby impacting its presence. The negative influence of PC_1_ reflects the topographical challenges in the northwestern region of Lemnos, primarily linked to the physical and ecological constraints of the terrain rather than the weather conditions. These areas often feature steeper slopes, reduced soil depth, and lower soil quality, all of which limit the suitable sites for burrow construction [47,127].

The spatial distribution patterns of *O. cuniculus* as extracted by KDE, the Getis-Ord Gi *, and Anselin Local Moran’s I provided important information into the hotspots’ intensity and clustering dynamics across the island. The density surfaces generated through KDE offered a foundational representation of the spatial distribution of *O. cuniculus*, with distinct peaks indicating areas of high rabbit presence. While these patterns revealed notable clusters, including a primary concentration in the central part of the island and 19 less dense regions across the study area (Figure 4), they did not conclusively determine whether these clusters were the result of randomness or underlying ecological processes [132].

To address this limitation, the Getis-Ord Gi * analysis not only validated the patterns observed in KDE but also provided robust statistical evidence, offering a more precise understanding of whether the observed clustering was statistically significant or the result of random variation [85]. The identified hotspots, supported by statistically validated clustering patterns, emphasized the central and northeastern parts of the island (Figure 5) as critical areas necessitating urgent conservation and management measures. The central hotspots align with fertile lowland plains characterized by a mosaic of cultivated and abandoned fields, which are interspersed with a few intermittently flowing streams bordered by fragmented riparian vegetation (pers. obs.). These areas provide abundant food sources and shelter for rabbits, making them highly suitable habitats. Approximately 30% of the land in this region remains uncultivated and unmanaged, primarily due to recurrent crop losses attributed to rabbit herbivory (pers. obs.). This land abandonment further enhances habitat suitability for *O. cuniculus*, as fallow fields promote the growth of dense vegetation, offering both forage and cover. Similarly, the northeastern hotspots are associated with cereal crop cultivation in flat, soft, and coastal terrain, which are highly favorable for burrowing [133]. The absence of significant slopes and the prevalence of cereal farming create productive environments that align with the species’ habitat preferences, contributing to the hotspots observed in this region.

In contrast, the coldspots in the central and northeastern parts of the island are located on highly eroded, shallow soils dominated by phrygana and maquis vegetation [59], as well as small forest patches of *Quercus ithaburensis* [58]. Although these areas exhibit some ecological similarities to the central hotspots, they are characterized by a lower agricultural intensity. The juxtaposition of these coldspots with the adjacent hotspots emphasizes the influence of microhabitat variability [134] and land-use practices on the spatial distribution of *O. cuniculus* [135].

The clustering patterns derived from Anselin Local Moran’s I analysis provide a granular understanding of the spatial distribution of *O. cuniculus* (Figure 6), offering a further refinement of the insights gained through KDE and Getis-Ord Gi *. The significant overlap of High–High (HH) clusters with conservation areas underscores the ecological importance of these regions, suggesting that conservation priorities must carefully balance habitat preservation with the need to manage rabbit populations, particularly to mitigate conflicts with agricultural activities. Moreover, the presence of Low–High (LH) clusters likely represents transitional zones or edge areas where habitat conditions are suboptimal for supporting high-density populations. These zones may also act as ecological buffers, reflecting gradients of habitat quality influenced by factors such as edge effects or varying land-use intensity.

Interestingly, no High–Low (HL) clusters were detected, which would represent isolated high-density areas surrounded by low-density areas. This absence suggests that high-density rabbit populations are embedded within larger, continuous clusters, highlighting the importance of spatial connectivity in sustaining robust populations of the species. The reliance of *O. cuniculus* on interconnected habitats underscores the role of landscape-scale factors, where environmental and anthropogenic influences converge to create favorable conditions. This spatial continuity, as evidenced by the lack of HL clusters, further emphasizes the need for integrated management strategies that maintain habitat quality across broader ecological networks, while addressing the processes driving population dynamics.

Expanding on this need for a landscape-scale perspective, the spatial lag model (SLM) offers deeper insights into the factors shaping hotspot intensity and clustering dynamics, emphasizing the interconnected nature of *O. cuniculus* populations on Lemnos Island. By accounting for spatial spillover effects, through the Rho coefficient, the SLM reveals how clustering patterns and hotspot intensities at one location are significantly influenced by the conditions in neighboring areas, with the spatial lag coefficient explaining a substantial portion of the variance (40.9% for hotspot intensity and 44.5% for clustering dynamics). This suggests that clustering patterns extend beyond localized environmental factors and reflects the presence of spatial spillover effects, as observed in studies applying spatial autoregressive models to investigate species distribution patterns, where the clustering patterns in one area influence those in adjacent locations [79,96,136,137]. This spatial dependency echoes the clustering patterns revealed by Anselin Local Moran’s I analysis, where High–High (HH) clusters demonstrated significant overlap with critical conservation areas. The observed spatial dependence does not imply direct species dispersal but rather reflects the spatial autocorrelation detected through the SLM, indicating that variations in hotspots and clustering dynamics are influenced by the spatial configuration of adjacent areas, suggesting the existence of underlying landscape-level processes due to shared environmental gradients and habitat connectivity [95,96]. This interconnectedness underscores the importance of viewing the species distribution not as isolated occurrences but as part of a broader spatial network, where habitat conditions and population density in adjacent areas collectively shape the species’ spatial structure across the island.

A particularly intriguing result is the negative relationship of PC_3_ (productivity potential) with both hotspot intensity and clustering dynamics in the SLM (Table 3), contrasting its positive association in the BLR model. This divergence likely reflects the scale-dependent complexity of ecological processes. At a local scale, as captured by the BLR model, increased productivity enhances habitat quality by providing abundant forage, thereby supporting the rabbit’s presence. However, at a broader spatial scale, as analyzed in the SLM, areas of excessive productivity may develop dense vegetation that limits movement, visibility, and the ability to construct burrows. This duality underscores the need to interpret productivity’s role within the specific spatial context in which it operates, as its effects can vary depending on whether the focus is on local habitat selection or broader landscape dynamics. Therefore, these findings reinforce the importance of adopting landscape-scale management strategies that integrate spatial spillover effects into conservation and mitigation planning. Effective management should prioritize targeted population control in high-density hotspots, particularly in agricultural areas, through well-documented methods such as culling [51,138], warren destruction [139], and exclusion fencing to protect crops [140], which have demonstrated success in reducing rabbit populations when combined with habitat management. Furthermore, the establishment of buffer zones around cultivated areas serves as an effective barrier to rabbit movement, mitigating human–wildlife conflicts [141].

However, approximately 60% of identified rabbit hotspots overlap with Natura 2000 conservation areas, necessitating an evaluation of the ecological trade-offs associated with these interventions. Culling within protected areas may disturb native fauna [142] through increased human presence and noise [143], disrupt predator–prey dynamics [144], and inadvertently impact non-target species [145]. Similarly, warren destruction could compromise soil integrity, disrupt plant communities, and affect species that are reliant on undisturbed habitats. Exclusion fencing, while non-lethal, may fragment habitats and alter wildlife movement patterns [146].

To mitigate these challenges, enhancing the populations of natural predators, particularly avian species such as buzzards, kestrels, and owls, presents a sustainable biocontrol option. This can be achieved through the creation of nesting sites in agricultural landscapes [147], providing safe breeding habitats that encourage predator presence. Additionally, integrating remote sensing technologies into management frameworks ensures the real-time monitoring of rabbit populations, enabling adaptive responses based on population dynamics [54]. Moreover, successful rabbit management on Lemnos Island hinges on collaborative efforts with local stakeholders, particularly farmers, whose participation ensures that management actions are not only scientifically sound but also socially supported and economically viable. By addressing the interconnectedness of habitats across Lemnos Island, such strategies can more effectively balance the needs of agricultural landscapes with ecological processes driving *O. cuniculus* distribution, ensuring the sustainable management of both rabbit populations and their habitats.

## 5. Conclusions

Our study provides the first spatially explicit baseline dataset for *Oryctolagus cuniculus* on Lemnos Island, contributing to a better understanding of the environmental and anthropogenic factors influencing its distribution. By integrating spatial methods, such as hotspot analysis, clustering metrics, and spatial lag modeling, we identified the complex interplay between environmental factors and spatial dependencies that influence the species distribution patterns.

Our results underscore the need for spatially informed management strategies that focus on mitigating *O. cuniculus* impacts in high-density areas while preserving broader landscape connectivity. The identified hotspots, which predominantly overlap with fertile lowland plains and conservation zones, highlight areas requiring immediate attention, and guiding measures such as habitat restoration, exclusion zones, and integrated pest management. Engaging local stakeholders, particularly farmers, in these strategies will ensure their long-term success and sustainability. By providing detailed spatial data and identifying areas of concern, we offer a foundation for informed decision-making to manage rabbit populations while balancing conservation and agricultural priorities. These insights may also inform strategies for similar conflicts in other insular ecosystems.

## Figures and Tables

**Table 1:** The PCA loadings and communality values of the five rotated principal components (PCs).

Principal Components	Variable	Principal Components Loadings				
		PC_1_	PC_2_	PC_3_	PC_4_	PC_5_
PC_1_—Elevation gradient	Minimum elevation	0.967	-	-	-	-
	Maximum elevation	0.958	-	-	-	-
	Mean Elevation	0.970	-	-	-	-
PC_2_—Arable and subsidized agriculture	Cereal crop cover	-	0.939	-	-	-
	Agricultural land cover	-	0.929	-	-	-
	State-supported agricultural area	-	0.871	-	-	-
PC_3_—Productivity potential	Minimum productivity	-	-	0.792	-	-
	Maximum productivity	-	-	0.870	-	-
	Mean productivity	-	-	0.969	-	-
PC_4_—Grazing-supporting landscapes	Total agricultural cover	-	-	-	0.884	-
	Pasture cover	-	-	-	0.751	-
PC_5_—Soil and field conditions	GAEC	-	-	-	-	0.933
	Soil depth	-	-	-	-	0.651

**Table 2:** The binary logistic regression model which illustrates the presence of *Oryctolagus cuniculus* on Lemnos Island. B = logistic coefficient; S.E. = standard error of estimate; Wald = Wald Chi-square; df = degree of freedom; *p*-value = significance.

Predictor	B	S.E.	Wald’s χ^2^	df	*p*-Value
PC_1_—Elevation gradient	−2.123	0.145	213.673	1	<0.001
PC_2_—Arable and subsidized agriculture	1.497	0.098	233.958	1	<0.001
PC_3_—Productivity potential	1.023	0.088	135.888	1	<0.001
PC_4_—Grazing-supporting landscapes	−2.519	0.152	275.567	1	<0.001
PC_5_—Soil and field conditions	4.404	0.176	624.502	1	<0.001
Constant	−0.380	0.111	11.723	1	<0.001

**Table 3:** Spatial lag model (SLM) analysis for the hotspot intensity and clustering dynamics of *Oryctolagus cuniculus* on Lemnos Island. Significant predictors are shown with their coefficients, standard errors (S.E.), z-values, and *p*-values. PC_1_ (elevation gradient) was not significant in the initial model and was excluded from the analysis.

Variable	B	S.E.	z-Value	*p*-Value
(a) SL-Hotspot Intensity Model				
W_ij_GiZscore_j_ (Spatial lag)	0.639	0.009	80.837	<0.001
Constant	0.344	0.044	7.770	<0.001
PC_2_—Arable and subsidized agriculture	0.154	0.034	4.497	<0.001
PC_3_—Productivity potential	−0.082	0.024	−3.404	<0.001
PC_4_—Grazing-supporting landscapes	−0.212	0.048	−4.367	<0.001
PC_5_—Soil and field conditions	0.073	0.029	2.463	<0.013
(b) SL-Clustering Dynamics Model				
W_ij_LMiZscore_j_ (Spatial lag)	0.667	0.008	92.391	<0.001
Constant	0.246	0.045	5.434	<0.001
PC_2_—Arable and subsidized agriculture	0.080	0.036	2.354	<0.01
PC_3_—Productivity potential	−0.073	0.025	−2.931	<0.01
PC_4_—Grazing-supporting landscapes	−0.076	0.051	−1.513	<0.05
PC_5_—Soil and field conditions	0.081	0.031	2.600	<0.001

## Data Availability

The raw data supporting the conclusions of this article will be made available by the corresponding author on request.
